# Development of an *ex vivo* human-porcine respiratory model for preclinical studies

**DOI:** 10.1038/srep43121

**Published:** 2017-02-24

**Authors:** Sophie Perinel, Jérémie Pourchez, Lara Leclerc, John Avet, Marc Durand, Nathalie Prévôt, Michèle Cottier, Jean M. Vergnon

**Affiliations:** 1 INSERM, U1059, SAINBIOSE, Saint-Etienne, F-42023, France; 2 Université de Lyon, Saint-Etienne, F-42023, France; 3 CHU Saint-Etienne, Saint-Etienne, F-42055, France; 4 Ecole Nationale Supérieure des Mines de Saint-Etienne, CIS-EMSE, SAINBIOSE, F-42023 Saint Etienne, France; 5 Centre Hospitalier Emile Roux, F-43012, Le Puy en Velay, France

## Abstract

Anatomical models to study aerosol delivery impose huge limitations and extrapolation to humans remains controversial. This study aimed to develop and validate an *ex vivo* human-like respiratory tract model easy to use and relevant to compare to *in vivo* human data. A human plastinated head is connected to an *ex vivo* porcine pulmonary tract ventilated artificially by passive expansion. A physiological study measures “pleural” depressions, tidal volumes, and minute ventilation for the respiratory rates chosen (10, 15, and 20 per minute) with three inspiratory/expiratory ratios (1/1, 1/2, and 1/3). Scintigraphy with ^81m^Krypton assesses the homogeneity of the ventilation. Forty different experiments were set for validation, with 36 (90%) ventilating successfully. At a respiratory rate of 15/minute with inspiratory/expiratory ratio of 1/2, the tidal volume average was 824 mL (standard deviation, 207 mL). The scintigraphy performed on 16 *ex vivo* models (44.4%), showed homogenous ventilation with great similarity to human physiological studies. Ratio of the peripheral to central count rates were equally correlated with human data published in the literature. This new model, combining research feasibility and human physiology likeness, provides a realistic approach to human inhalation and therefore can be an interesting tool in aerosol regional deposition studies.

Inhalation is the main route of environmental particle exposure. It also represents an attractive pathway for local medications, such as bronchial diseases treatments, chemotherapy^1^, antibiotics^2^, and systemic medications, such as vaccinations, insulin therapy, and gene therapy^3^. To relate airborne particle exposure to wanted or unwanted biological effects, it is essential to assess regional deposition within the respiratory tract. Identification of deposition sites is a major determinant of particle bio-persistence, dose in tissues, and resultant biological effects.

Computational models allow calculation of inhalation dosimetry of particles^4^,^5^, but there is an experimental data gap for critical parameters (submicron-sized particles, specific anatomical features). This lack of dataset restricts the calibration or verification of mathematical prediction. Thus, experimental studies dedicated to regional particle deposition within the airways remain a key issue in successfully predicting sites of pathological changes and biological effects of inhaled medications. Three main types of *in vivo* aerosol studies can be performed: *in vivo* human studies; *in vivo* aerosol deposition studies on rodents; and *in vivo* aerosol deposition studies on pigs and non-human primates.

*In vivo* human studies are scarce because of ethical restrictions due to radiation exposure as regional deposition is usually assessed using inhalation of radio-labeled aerosols^6^. *In vivo* aerosol deposition studies using rodents are frequent^7^,^8^. However, these models are different from the human airways in size, bronchial divisions, and anatomy of upper airways^9^. For example, rodents have no lobe divisions in the left lung^10^ or the bronchial divisions are very different from humans in most part of mammals^11^. Moreover, physiological ventilation is very different. For example, respiratory rate at rest is approximately 80 per minute for a rat versus 15 per minute for an adult human. These differences are widely recognized as very significant on deposition profiles, so extrapolations from these models to humans must be done very carefully. *In vivo* aerosol deposition studies using pigs and non-human primates can be useful because of their anatomical likeness to human airways^12^,^13^,^14^. For example, non-human primates such as baboons are used to mimic the respiratory tract of children^15^,^16^,^17^. Although these studies appear very relevant, important limitations remain such as ethical restrictions, high cost of experiments, and uncontrolled breathing pattern (inspired to expired ratio, frequency, obstruction, etc.) in spontaneous breathing.

Finally, bridging the existing gap on respiratory models to assess aerosol regional deposition on human adults, this study aimed to develop an *ex vivo* human-like respiratory tract model with controlled breathing parameters allowing simulation of numerous physiopathological conditions. It also aimed to develop a less expensive *ex vivo* preclinical model that is easy to use and that emphasizes no ethical restriction and high relevance compared to *in vivo* human data. This new concept is composed of a human plastinated head connected to an *ex vivo* porcine pulmonary tract ventilated artificially by passive expansion with pleural depressions. The main objective of this work was validating this model in comparison with human physiology and other existing models. More specifically, this study assesses the experimental development of the respiratory model, the physiologic characteristics of its ventilation, the influence of respiratory variations and the homogenous of the ventilation by scintigraphy.

## Methods

### Materials

**Figure 1:**
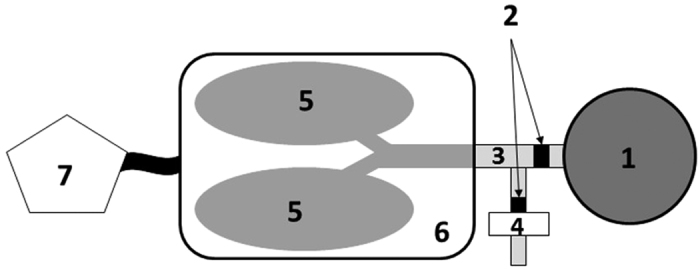
*Ex vivo* chimeric preclinical model: schematic representation. (1) Human plastinated head, (2) one-way valves, (3) plastic tubes, (4) expiratory filter, (5) porcine pulmonary tract (intrathoracic), (6) plastic box and (7) respiratory pump. A picture is available as online Supplement Figure S2.

Plastination is a technique of preservation that allows an anatomical and physical state approaching that of live physiological conditions. The specimen was obtained from a deceased man whose last will and testament documented the wish to leave his body to the Saint Etienne Anatomy Laboratory in accordance with the law and ethics committee (informed consent). Anatomic and aerodynamic investigations demonstrated behavior of the human cadaver plastinated head (Supplementary Figure S1) in accordance with a healthy subject with nasal decongestion^18^,^19^,^20^. This cast is well-adapted and already used for functional studies such as nasal flow, drug delivery, and aerosol deposition studies^21^,^22^. The laryngeal part (Fig. 1) is made of plastic tubes with a one-way valve simulating the resistance of vocal folds.

The intra-thoracic (IT) part of the respiratory tract is obtained from porcine slaughterhouses, satisfying all the sanitary controls in accordance with sanitary security, and is used within 48 hours (except if there is a freezing process). All experimentations are performed according to the best practice guidelines of laboratory animal exploitation^23^ and according to national and American recommendations. A bronchoscopy is systematically performed and all the observations are recorded (obstructions, secretions, need for instillation, bronchial divisions etc.). The IT part is ventilated using a specific device (Super Dimension^®^; Covidien, Dusseldorf Germany) consisting of an instrumented sealed enclosure (Fig. 1) specifically developed to simulate *in vivo* ventilation by pleural depression. A picture is available as online supplementary Figure S2.

### Methods

The depressions (negative pressures approximately 8 to 10 kPa) performed in the sealed enclosure are continuously measured using a differential pressure transmitter (Autotran 860; Mesureur society, Chilly-Mazarin, France). The real-time air speed is recorded in the trachea using an anemometer (MINIAIR6 mini; Mesureur society). These data allow calculation of the air output in the trachea representing the minute ventilation. The tidal volume (VT) is obtained by integration for each respiratory cycle. An average of VT and minute ventilation is calculated for several respiratory cycles in each specimen of the model. These data are recorded with breathing rates of 10, 15, and 20 per minute, with inspiratory/expiratory (I/E) ratios of 1/1, 1/2, and 1/3 for each rate. The breathing parameters are chosen to represent variations of adult human physiology at rest^24^,^25^,^26^. Due to the absence of the rib cage, the end expiratory lung volume is not stable and consequently cannot be determined reliably.

Planar ventilation scintigraphy is performed using ^81m^Krypton (^81m^Kr)^27^,^28^ at ambient temperature. For each *ex vivo* model, an acquisition is made for each respiratory rate (10, 15, and 20/min) with a naso-buccal mask. Three different regions of interest are identified on ventilation scintigraphy to define left lung and right lung as well as central area and peripheral area (by a rectangle of 20% and 50%, respectively, of each lung)^29^,^30^. Additional details about the method are provided in the online data supplement.

### Statistical analysis

Results are reported as numbers (%) or average (standard deviation). Continuous variables were compared using the non-parametric two-way ANOVA and the non-parametric Tukey’s multiple comparisons tests. All tests were two-sided and *P* < 0.05 was considered statistically significant. The physiological data are analyzed with NextView^®^ 4 Lite software (BMCM, Maisach, Germany). Statistical analyses were performed using Excel 2010 (Microsoft Office^®^, Redmond, WA, USA) and GraphPad Prism^®^ 6 (GraphPad Software, La Jolla, CA, USA).

## Results

### Feasibility

The validation of the *ex vivo* model was performed using 36 different porcine IT tracts connected to the same human plastinated head. All porcine IT tracts were used within 24 hours after slaughter, except three (8.3%) that were frozen and thawed because of supplier unavailability. The experimentation has been performed 40 different times (90% successfully); four models were excluded because three preliminary models were not satisfactorily ventilated and one had too many cuts. A total of 2 hours was necessary to set up the whole model, after which it was able to be used for the entire day. The model was never conserved more than 1 day for sanitary reasons. The weight of the IT tract ranged from 619 g to 1039 g (median, 760 g; interquartile range [IQR], 147.5). A median of 9.5 stitches per IT tract was realized (IQR, 12), and the median length of trachea was 7 cm (IQR, 1). Except for aspirations, flexible bronchoscopies were necessary for seven (19.4%) IT tracts; the airways were cleaned with a saline solution. A video (Supplementary Video SV1) of this model is provided in the online data supplement.

### Anatomical and physiological features

**Figure 2:**
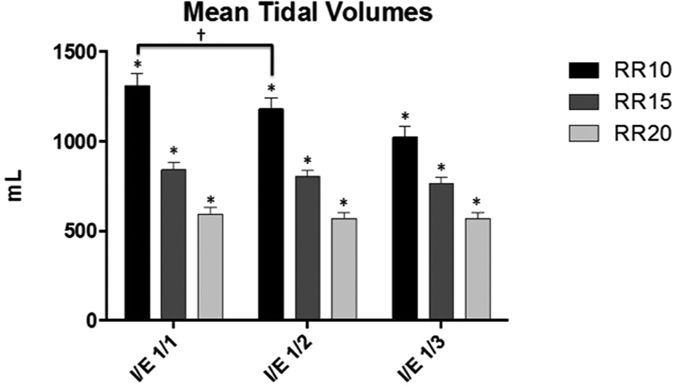
Comparison of average of tidal volumes. VT: tidal volume (mL); RR: respiratory frequency; I/E: inspiratory/expiratory ratio. *p < 0.001.

The *ex vivo* model allows variations of inspiratory and expiratory times. Consequently, the respiratory rate and I/E ratios can be chosen as necessary for experimentation. In this study, the physiological features have been determined for the three respiratory rates and I/E ratios chosen. Each value of VT has been calculated by averaging several cycles (median, 5; IQR, 1). The average of maximal depressions measured in the box ranged from −1.42 to −3.68 kPa. Table 1 shows the VT, the maximal depressions, and the minute ventilation for every ventilation parameter, along with standard deviation and variance. Figure 2 shows the comparison between the different physiological parameters. With a constant I/E in each group, VT statistically significantly decreases as the respiratory frequency increases (*p* < 0.001 for every comparisons). For the same respiratory rate, VT is not significantly different when there is a change in I/E, except for respiratory rate of 10/min with an I/E of 1/1. For all calculations of average VT, the intrinsic variance comprises between 0% and 12.2% Any differences were observed for lungs used after freezing process.

### Scintigraphic measurement

**Figure 3:**
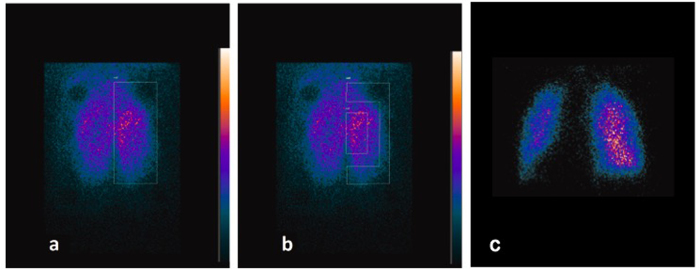
81mKrypton planar scintigraphic images with the regions of interest (ROIs). (**a**) Left ROI. (**b**) Central (20% of the lung) and peripheral (50% of the lung) ROIs. (**c**) A human planar scintigraphy of normal lungs from the evaluation of a patient in the ward leading to the conclusion of a normal lung function.

The ^81m^Kr ventilation scintigraphy has been performed on 16 *ex vivo* models (44.4%), and each time the ^81m^Kr generator was available. The two different types of regions of interest are shown in Fig. 3. Ratios of the count rates between peripheral to central regions and of left lung to the total count for both lungs are calculated to compare with human studies using these original markers (20, 24, 25). First, the ratio of the left lung to the total count rate is calculated; it ranges from 43.3% to 59.2% (median, 49.7%; IQR, 4.6%). Second, the ratio of the count rates between peripheral to central regions is calculated; it ranges from 0.541 to 0.767 (median, 0.616; IQR, 0.08). The comparison of results between the three groups showed no significant differences in ratios according to the different respiratory rates and I/E.

## Discussion

To the best of our knowledge, this is the first preclinical respiratory model allowing the assessment of aerosol regional deposition with data-gathering feasibility, excellent reproducibility, and human anatomic similarity. This model is an ethical and inexpensive alternative to *in vivo* laboratory animal experiments. Moreover, a main advantage of this new concept is that it accurately controls the ventilation parameters and fits the human physiology. This could provide fundamental insights because, as noted for aerosol therapy, inhalation studies mainly depend on the respiratory pattern for regional deposition (*i.e.*, ENT versus pulmonary deposition)^31^.

All the physiological features (tidal volume, respiratory rate, etc.) are consistent with the widely accepted human physiology for an adult at rest, as found in medical textbooks^32^, confirming the “human-like” features. Moreover, all experimental conditions are highly biomimetic, such as the original breathing technological process simulating the intrapleural depression. Because its half-life after death is a few hours, surfactant is absent in the *ex vivo* porcine lung. This had to be taken into account in our experimental conditions. The simulated intra-pleural depressions necessary to inflate the lungs in the sealed enclosure were adjusted to compensate for the absence of surfactant. As a result, the model requires twice the values of depression usually obtained for human breathing, which is consistent with the role of the surfactant (approximately 50% compliance)^32^. It has to be noted that the absence of circulation in this model impacts the compliance too. Even though regional deposition data in humans have been developed as a function of particle size as early as the 1960s, some experimental aerosol deposition experiments in humans and laboratory animals focused on the assessment of the total deposited fraction^33^,^34^,^35^,^36^. These studies agreed on the major influence of the anatomy and the ventilation parameters on deposition. The respiratory tracts of pigs are very similar to those of humans, with the same 23 branching divisions except for position of the tracheal bronchus, making pigs very good animal models of the human respiratory tract for decades^37^. Therefore, this model achieves the major functional and anatomical qualities necessary to study airborne deposition without the drawbacks of *in vivo* studies (ethical restriction, need for expensive infrastructures).

The ^81m^Kr ventilation scintigraphy has been widely used as a highly interesting medical examination of lung function for more than three decades. Because of its very short half-life (13 sec), its distribution is considered proportional to the regional ventilation of the lungs^28^. This part of the study has been designed to be consistent with the original principles published by Fazio *et al*.^29^. The ratio of count rates of peripheral to central regions of interest (called penetration index) and their definitions are similar to comparable results (0.509 to 0.696; median, 0.625; IQR, 0.07 for the normal subjects in the study by Fazio). Moreover, Fazio has pointed out the heterogeneous images obtained with pathological subjects such as obstructive patients^27^. As can be observed in Fig. 3, this is not the case with our model; therefore, this seems to be another element confirming the interesting ventilation capacity of our model. For the regional deposition using scintigraphy, this work has been designed as previous works in order to have a model allowing aerosol studies comparable to literature^38^,^39^.

Some limitations of this model have to be noted. First, the most important and incurable limit is the same as that of any other model: it will never be equivalent to the human *in vivo* study with spontaneous ventilation. However, such models are needed for practice. The supine position decreases the gradient of penetration of the particles in the lungs^40^; therefore, this could be a limitation in our model because other positions are impossible. Moreover, due to the absence of the rib cage, the end expiratory lung volume is not stable and cannot be reliably determined. Consequently, hyperinflation could occur with regional ventilation inequalities. Nevertheless, the scintigraphic study macroscopically assesses the regional ventilation and its homogeneity. Also, the plastinated head used only permits nasal breathing, which is usually not the exclusive way of breathing for adults^31^. The model could be improved regarding this point, and further research is in progress to correct this limit. For the scintigraphic study, the ratio of the left lung to the total count rates for both lungs (43.3% to 59.2%; median, 49.7%; IQR, 4.6%) is different from that of original publications using humans (48%) and from the study of Möller *et al*.^41^ (average, left/right ratio 0.90; standard deviation, 0.06). The lack of left–right asymmetry could be explained by the absence of the rib cage; consequently, the lungs lie flat with a median position for the middle lobe, which is usually part of the right lung. Finally, future inhalation studies of this new model will need to compare the results to the predictions of the widely used mathematical models^5^.

Besides its originality, this *ex vivo* human-like preclinical model has several strengths. Compared to *in vivo* data, it allows less expensive experiments with no ethical restrictions because any animal is specifically sacrificed for this model. The adjustment of inspiratory and expiratory times allows the respiratory frequency and the ratio between inspiration and expiration to be chosen. Consequently, many physiological or pathological conditions can be studied, especially airway obstructions. The averages of VT and their correlation to respiratory frequency are consistent with the physiology of an adult at rest^42^. There is also excellent intrinsic reproducibility, as the VT variance is less than 12% for each model. Because the velocity of airborne particles affects their probability of impacting the oropharynx and larynx, the control of airflow is crucial. It is widely known that a maximum of 30 L/min is an ideal condition for inhalation of adults at rest with a jet nebulizer^31^, and the model fits this point.

Finally, most aerosol regional depositions are currently based on mathematical models. Our study does not aim to replace *in silico* predictions nor *in vivo* human studies and does not pretend to be comparable. However, for the first time, there is a less expensive model that is easy to use and that has a realistic approach to human inhalation. Therefore, our model is an interesting tool for aerosol deposition studies because it suits the research requirements while being as close as possible to human physiology and limiting live animal experiments.

## Additional Information

**How to cite this article**: Perinel, S. *et al*. Development of an *ex vivo* human-porcine respiratory model for preclinical studies. *Sci. Rep.* **7**, 43121; doi: 10.1038/srep43121 (2017).

**Publisher's note:** Springer Nature remains neutral with regard to jurisdictional claims in published maps and institutional affiliations.

## Supplementary Material

Supplementary Information

Supplementary Video SV1

## Figures and Tables

**Table 1:** Physiological data recorded on the model.

I/E	RR 10			RR 15			RR 20		
	1/1	1/2	1/3	1/1	1/2	1/3	1/1	1/2	1/3
**Tidal volume**									
Average (mL)	1335	1189	1026	859	824	764	604	579	585
Standard deviation	374	335	350	240	207	193	218	175	181
Variance (%)	28.0	28.2	34.1	27.9	25.1	25.2	36.1	30.3	31.0
**Measured depressions**									
Average (kPa)	−3.68	−2.61	−1.94	−3.39	−2.32	−1.65	−3.17	−1.90	−1.42
Standard deviation	1.25	0.73	0.43	1.21	0.87	0.51	1.15	0.64	0.49
Variance (%)	−33.9	−27.9	−22.1	−35.6	−37.6	−31.1	−36.4	−33.7	−34.7
**Ventilation per minute**									
Average (L/min)	31.24	36.45	37.33	28.71	35.06	37.59	25.26	32.29	35.15
Standard deviation	9.46	12.69	16.41	7.97	11.45	14.19	8.06	10.87	14.34
Variance (%)	18.2	20.9	26.4	16.7	19.6	22.6	19.2	20.2	24.5

RR: respiratory rate; I/E: inspiratory/expiratory ratio.
