# Supplementary material for: Development of an ex vivo human-porcine respiratory model for preclinical studies
Source: Sci Rep. 2017 Feb 24;7:43121. doi: 10.1038/srep43121 (PMC5324051; doi:10.1038/srep43121)
Supplement: Supplementary Information [file srep43121-s1.doc]

**Development of an *ex vivo* human-porcine respiratory model for preclinical studies**

Sophie Perinel, Jérémie Pourchez, Lara Leclerc, John Avet, Marc Durand, Nathalie Prevot-Bitot, Michèle Cottier, Jean M. Vergnon.

**Supplementary material for online supplement**

Materials

Experiments were performed using a human plastinated head obtained using a specific plastination technique (E1, E2). The specimen was obtained from a deceased man who donated his body to the Saint Etienne Anatomy Laboratory in accordance with the law and ethics committee (Figure S1).

Plastination allows anatomical specimens to be preserved in a physical state approaching that of live physiological conditions with numerous advantages (E3). The technical specifications of the plastinated cast developed were: anatomical features as close as possible to *in vivo* human airways; time stability for several years; water-washable; easy daily handling; dry and odorless; biologically safe; and transportable without restrictions (E4). Moreover, the cast used for this study has a high level of anatomic quality, including very good mucosa preservation. Aerodynamic and geometrical investigations using several techniques (endoscopy, CT scans, acoustic rhinometry, and rhinomanometry) highlighted global behavior in accordance with a healthy subject with nasal decongestion (E1, E2).

The laryngeal part is made of plastic tubes with an internal section of 1.7 cm and a one-way valve simulating the resistance of vocal folds. The tracheal length is the closest to human physiology (approximately 12 cm) and is systematically recorded. Exhalation is allowed by another one-way valve with an expiratory filter for the retention of particles, differentiating the exhaled fraction of particles inhaled from the fraction persisting inside the organism.

The intra-thoracic (IT) part of the respiratory tract is obtained from porcine slaughterhouses, satisfying all the sanitary controls and working in accordance with French law and sanitary security. All experimentations are performed according to the best practice guidelines of laboratory animal exploitation (E5) and according to national and American recommendations. The pigs have been slaughtered within 24 hours, and precise dissection is performed just before experimentation to keep only the lungs and the trachea, specifically avoiding wounds. Finally, the IT respiratory tract is washed with water, weighted, and inserted in a plastic box that is hermetically closed and linked to the generator.

A bronchoscopy is systematically performed and all the observations are recorded. The IT part is ventilated using a specific device (Super Dimension®; Covidien, Dusseldorf Germany) that allows simulation of *in vivo* ventilation by pleural depression, thanks to negative pressures generated (approximately 8 to 10 kPa) in the sealed enclosure containing the porcine respiratory tract (E6). Video E1 is a short video of the model as ventilation is occurring.

Methods

The depressions in the sealed enclosure around the lungs (i.e., the simulated “pleural cavity”) are continuously measured thanks to a differential pressure transmitter (range, 0–200 mBar; Autotran 860; Mesureur society, Chilly-Mazarin, France). The real-time air speed is recorded in the trachea thanks to an anemometer (range, 0.3–20 m/s; MiniAir6 mini; Mesureur society, Chilly-Mazarin, France), placed just after the one-way valve simulating the larynx. These data allow the calculation of the air output in the trachea, representing the minute ventilation. The tidal volume is obtained by integration for each respiratory cycle. Average tidal volume and minute ventilation are calculated for several respiratory cycles for each specimen of the model. These data are recorded with breathing rates of 10, 15, and 20 per minute and inspiratory/expiratory ratios of 1/1, 1/2, and 1/3. The breathing parameters chosen correspond to adult physiology at rest, with three samples studied for each experiment to take into account possible variations in human physiology (E7–E9). Due to the absence of the rib cage, the end expiratory lung volume is not stable and consequently cannot be determined reliably.

Planar ventilation scintigraphy is performed using ^81m^Krypton (^81m^Kr), depending on ^81m^Kr generator availability (E10, E11). This radioactive gas (rubidium (81 Rb)/^81m^Kr) generator; Covidien Imaging, Elancourt, France) is continuously administered to the model through a facial mask with an oxygen output of 8 L/min, according to the security protocol of the nuclear medicine department of Saint Etienne University Hospital and recent guidelines (E11). The images are recorded with a rectangular, single-head nuclear medicine camera (Millennium MPR Gamma Camera; GE Healthcare, Saint Cyr au Mont D’or, France) equipped with a low-energy, high-resolution collimator. For each *ex vivo* model, three anterior planar scintigraphies are successively acquired (matrix 128*128) during gas ventilation for each respiratory rate (10, 15, and 20/min) with a target of 200,000 counts per acquisition. Three different regions of interest are identified on ventilation scintigraphy to define left lung and right lung as well as central area and peripheral area (by a rectangle of 20% and 50%, respectively, of each lung) (E12, E13). The analysis of scintigraphy has been made by ratios of counts per pixel for each acquisition with calculation of the total ratio of the lungs and left, right, central, and peripheral ratios using a Xeleris 2 workstation (GE Healthcare). Then, a ratio analysis is performed (peripheral/central and left/total) to compare with human studies using these original markers (E10, E14, E15).

Statistical analysis

Results are reported as numbers (%) or average (standard deviation). Continuous variables were compared using a two-way ANOVA and the non-parametric Tukey’s multiple comparisons test. All tests were two-sided and *P* < 0.05 was considered statistically significant. The physiological data were analyzed with NextView® 4 Lite software (BMCM, Maisach, Germany) and then extracted. Statistical analyses were performed using Excel® 2010 (Microsoft Office, Redmond, WA, USA) and GraphPad Prism® 6.

Results

For the scintigraphic assessment, left, right and total count rates are shown in Figure S3 and the central and peripheral count rates are shown in Figure S4.

**References**

E1. Croce C, Fodil R, Durand M, Sbirlea-Apiou G, Caillibotte G, Papon J-F, Blondeau J-R, Coste A, Isabey D, Louis B. In vitro experiments and numerical simulations of airflow in realistic nasal airway geometry. Ann Biomed Eng 2006;34:997–1007.

E2. Durand M, Rusch P, Granjon D, Chantrel G, Prades JM, Dubois F, Esteve D, Pouget JF, Martin C. Preliminary study of the deposition of aerosol in the maxillary sinuses using a plastinated model. J Aerosol Med Off J Int Soc Aerosols Med 2001;14:83–93.

E3. Von Hagens G. Impregnation of soft biological specimens with thermosetting resins and elastomers. Anat Rec 1979;194:247–255.

E4. Durand M, Pourchez J, Louis B, Pouget JF, Isabey D, Coste A, Prades JM, Rusch P, Cottier M. Plastinated nasal model: a new concept of anatomically realistic cast. Rhinology 2011;49:30–36.

E5. NIH OACU. Regulations and standards. Available from: http://oacu.od.nih.gov/regs/.

E6. Zarogoulidis P, Chatzaki E, Porpodis K, Domvri K, Hohenforst-Schmidt W, Goldberg EP, Karamanos N, Zarogoulidis K. Inhaled chemotherapy in lung cancer: future concept of nanomedicine. Int J Nanomedicine 2012;7:1551–1572.

E7. Booker R. Interpretation and evaluation of pulmonary function tests. Nurs Stand R Coll Nurs G B 1987 2009;23:46–56; quiz 58.

E8. Criée CP, Sorichter S, Smith HJ, Kardos P, Merget R, Heise D, Berdel D, Köhler D, Magnussen H, Marek W, Mitfessel H, Rasche K, Rolke M, Worth H, Jörres RA, Working Group for Body Plethysmography of the German Society for Pneumology and Respiratory Care. Body plethysmography—its principles and clinical use. Respir Med 2011;105:959–971.

E9. Flesch JD, Dine CJ. Lung volumes: measurement, clinical use, and coding. Chest 2012;142:506–510.

E10. Fazio F, Jones T. Assessment of regional ventilation by continuous inhalation of radioactive krypton-81m. Br Med J 1975;3:673–676.

E11. Bajc M, Neilly JB, Miniati M, Schuemichen C, Meignan M, Jonson B, EANM Committee. EANM guidelines for ventilation/perfusion scintigraphy : Part 1. Pulmonary imaging with ventilation/perfusion single photon emission tomography. Eur J Nucl Med Mol Imaging 2009;36:1356–1370.

E12. Fazio F, Wollmer P, Lavender JP, Barr MM. Clinical ventilation imaging with In-113m aerosol: a comparison with Kr-81m. J Nucl Med Off Publ Soc Nucl Med 1982;23:306–314.

E13. Amis TC, Crawford AB, Davison A, Engel LA. Distribution of inhaled 99mtechnetium labelled ultrafine carbon particle aerosol (Technegas) in human lungs. Eur Respir J Off J Eur Soc Clin Respir Physiol 1990;3:679–685.

E14. Hannan WJ, Emmett PC, Aitken RJ, Love RG, Millar AM, Muir AL. Effective penetration of the lung periphery using radioactive aerosols: concise communication. J Nucl Med Off Publ Soc Nucl Med 1982;23:872–877.

E15. Möller W, Meyer G, Scheuch G, Kreyling WG, Bennett WD. Left-to-right asymmetry of aerosol deposition after shallow bolus inhalation depends on lung ventilation. J Aerosol Med Pulm Drug Deliv 2009;22:333–339.

**Figures legends**

**Video SV1: Video of the model as ventilation is occurring.**

**
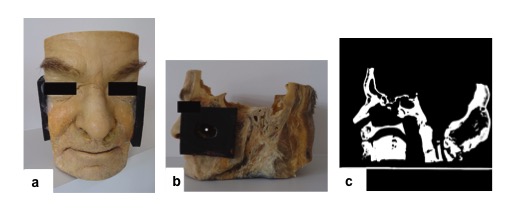
**

**Figure S1: The plastinated head (a and b) and its CT scans (c).** **a:** Frontal view of the plastinated head. **b:** Lateral view of the plastinated head (*opening of the maxillary sinuses hermetically closed during all the experiments). **c**: Vertical scan of the head.


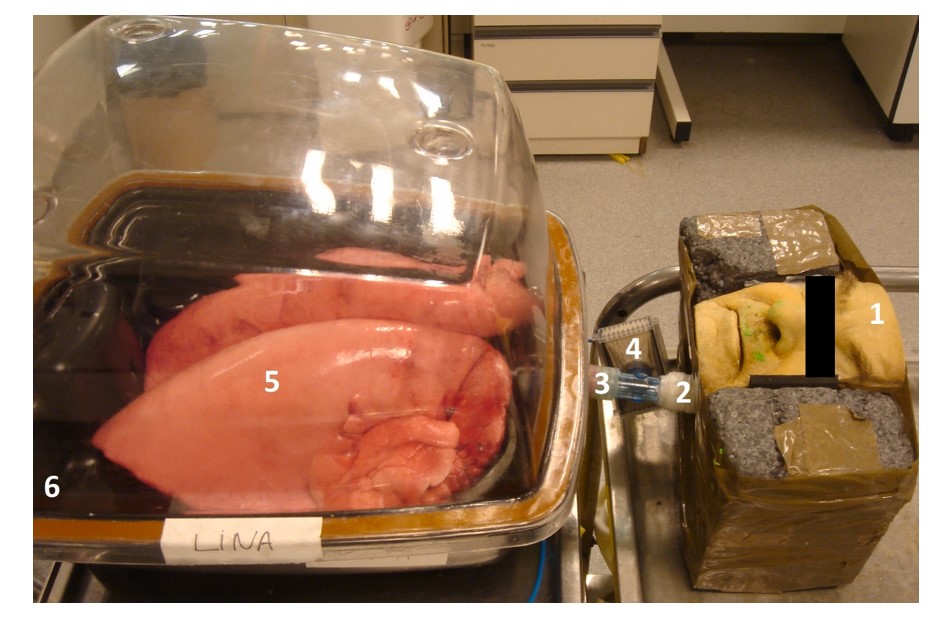


**Figure S2:** ***Ex vivo* chimeric preclinical model: picture of the model.** (1) Human plastinated head, (2) one-way valves, (3) plastic tubes, (4) expiratory filter, (5) porcine pulmonary tract (intrathoracic), (6) plastic box.


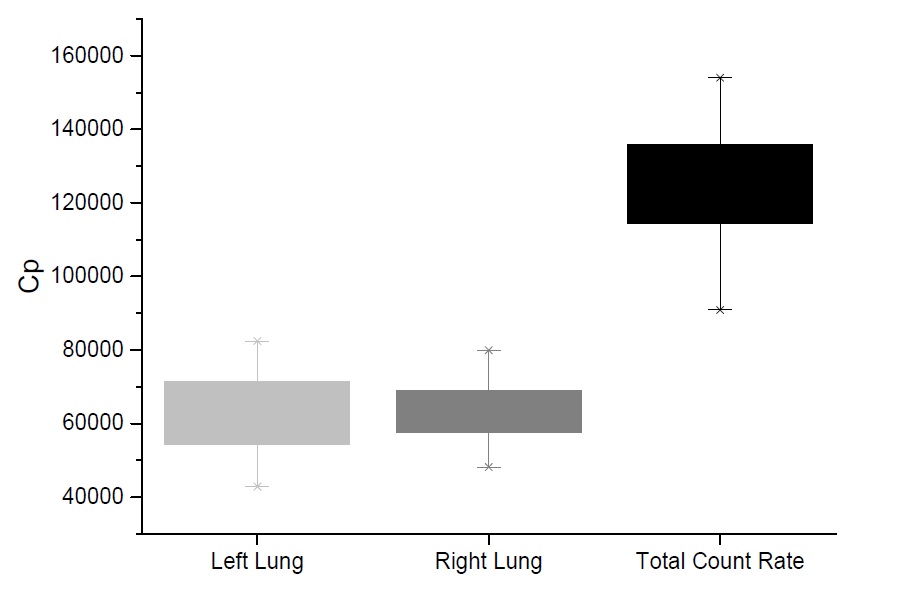


**Figure S3: Left, right and total count rates of Krypton scintigraphy**

Cp : count

**
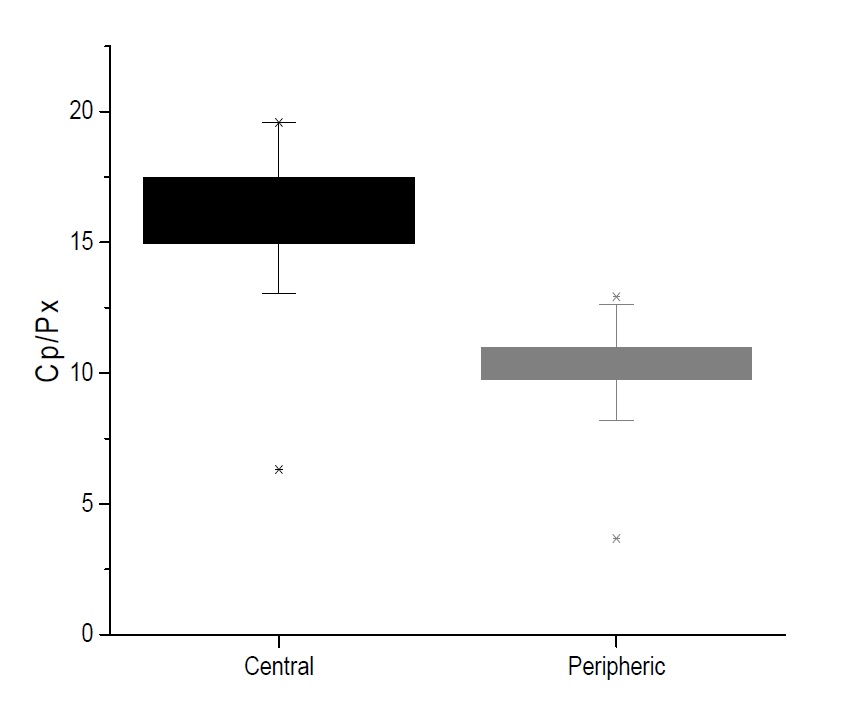
**

**Figure S4: Central and peripheral count rates ratios**

Cp/px : count per pixel
